# DXA-Derived Body Composition and Insulin Resistance at Preschool Age in Very-Low-Birth-Weight Preterm Infants: A Prospective Cohort Study

**DOI:** 10.3390/diagnostics16131991

**Published:** 2026-06-26

**Authors:** Kai-Ti Tseng, Chia-Huei Chen, Jui-Hsing Chang, Chyong-Hsin Hsu, Chia-Ying Lin, Wei-Hsin Ting, Ya-Ting Jan, Hung-Yang Chang

**Affiliations:** 1 Department of Pediatrics, MacKay Children’s Hospital, Taipei 104217, Taiwan; kt.4980@mmh.org.tw (K.-T.T.);; 2 Department of Medicine, MacKay Medical University, New Taipei City 251020, Taiwan; 3 Institute of Emergency and Critical Care Medicine, College of Medicine, National Yang Ming Chiao Tung University, Taipei 112304, Taiwan; 4 Department of Radiology, MacKay Memorial Hospital, Taipei 104215, Taiwan

**Keywords:** body composition, fat, insulin resistance, preterm, very low birth weight

## Abstract

**Background/Objectives:** Preterm infants have higher fat mass and lower lean mass at term-corrected age; however, whether these differences persist into preschool age remains unclear. This prospective observational cohort study aimed to compare body composition between very-low-birth-weight (VLBW) preterm (gestational age < 33 weeks) children and their term-born counterparts aged 5–6 years. **Methods:** Anthropometric data, body composition, blood biochemical parameters, and insulin resistance (HOMA-IR index) were compared between the preterm and term groups. **Results:** The study included 96 children (57 preterm and 39 term-born). Although lean mass index and fat mass index were comparable between groups, preterm children exhibited significantly higher insulin levels and HOMA-IR values after adjustment (*p* = 0.003 and *p* = 0.004, respectively). Within the preterm cohort, overweight/obesity was associated with higher trunk and total fat percentages, as well as higher HOMA-IR, compared with those of normal-weight or underweight children (all adjusted *p* < 0.001). Weight growth velocity from 2 to 5 years was positively associated with serum insulin, HOMA-IR, and both trunk and total body fat percentages. Additionally, girls in both groups displayed significantly higher trunk and total body fat percentages than boys. **Conclusions:** Children born very preterm with VLBW had higher fasting insulin levels and HOMA-IR, despite generally comparable DXA-derived LMI, FMI, and fat distribution at preschool age. Overweight status and rapid early childhood weight gain may contribute to increased metabolic risk in this population, highlighting the need for early metabolic monitoring and growth management. Future large-scale, long-term studies are required to confirm these findings.

## 1. Introduction

Despite improved survival rates among very-low-birth-weight (VLBW) infants, the long-term consequences of prematurity remain a concern. Although prior research has primarily focused on growth and neurodevelopmental outcomes, accumulating evidence links preterm birth to broader cardiometabolic risk, including hypertension, cardiovascular dysfunction, metabolic syndrome, and chronic kidney disease [1,2,3,4,5,6].

Preterm infants exhibit distinct differences in body composition during early postnatal life [7]. A systematic review reported that preterm infants have a higher fat percentage and lower fat-free mass than their term-born peers at term-equivalent ages [7]. However, reports on subsequent body composition changes remain inconclusive. Some studies suggest that preterm children continue to exhibit a higher percentage of body fat into preschool age and childhood [8], whereas others have found no significant differences compared with term-born controls [9,10]. This inconsistency may partly reflect heterogeneity in maternal and neonatal characteristics. Factors such as genetic background, sex, perinatal stress, maternal diabetes, nutritional status, and neonatal morbidities may jointly influence body composition, insulin sensitivity, and the risk of later non-communicable diseases via complex and interrelated pathways [11,12,13].

Early nutritional exposure and postnatal growth may further modify later body composition and metabolic health in preterm infants [2]. Infants born prematurely who are small for gestational age (SGA) or have intrauterine growth restriction (IUGR) may be particularly vulnerable to adverse metabolic programming and increased intra-abdominal fat accretion during subsequent catch-up growth. In addition, traditional nutritional strategies aimed at promoting rapid weight gain through hypercaloric feeding may contribute to accelerated weight gain and disproportionate fat accumulation. Feeding practices may also influence later body composition, as the use of standard formula rather than fortified human milk has been associated with greater fat deposition relative to fat-free mass in some studies. Although early fat accretion may be physiologically advantageous by supporting thermoregulation, energy storage, and neurodevelopment, rapid postnatal weight gain has also been linked to adverse long-term metabolic outcomes. In particular, accelerated growth during later infancy or childhood has been associated with greater adiposity, insulin resistance, hypertension, and cardiovascular risk [11,12,13,14,15,16]. Furthermore, preterm infants with IUGR may subsequently experience extrauterine growth restriction, potentially further affecting long-term growth and metabolic outcomes. Nevertheless, the long-term relationships among prematurity, early growth, body composition, and metabolic health remain incompletely understood, particularly in preschool-aged children born very preterm with VLBW.

In addition to neurodevelopmental impairment, previous studies have reported abnormalities in pulmonary, cardiovascular, and renal outcomes among VLBW survivors during the preschool years [2,3,4,5]. However, data regarding body composition and metabolic health at this age remain limited. Furthermore, the preschool period provides an opportunity to evaluate body composition and metabolic outcomes before the hormonal changes in puberty substantially influence these parameters. We therefore hypothesized that by preschool age, children born before 33 weeks of gestation with VLBW would exhibit altered fat distribution and greater insulin resistance compared with term-born controls. The primary aim of this study was to evaluate body composition using dual-energy X-ray absorptiometry (DXA), together with biochemical markers, in children aged 5–6 years who were born before 33 weeks of gestation with VLBW compared with those of term-born controls. A secondary aim was to explore the associations of perinatal factors, nutritional exposures, and postnatal growth patterns with body composition and metabolic outcomes in the preterm group.

## 2. Materials and Methods

### 2.1. Study Population

This prospective longitudinal cohort study included children aged 5–6 years who were born at <33 weeks of gestation with VLBW and attended follow-up visits at MacKay Children’s Hospital, Taipei, Taiwan, between 1 January 2021 and 31 December 2022. The age of 5–6 years was selected as the primary assessment time point because it represents a standardized follow-up milestone within our institutional VLBW follow-up program. Age-matched term-born children with normal body mass index (BMI) were recruited from the hospital’s healthy child vaccination clinic as the control group. Children with chromosomal abnormalities or congenital anomalies were excluded. This study was financially supported by a grant MMH-109-82 from MacKay Memorial Hospital. The study protocol was approved by the Institutional Review Board of MacKay Memorial Hospital (approval no. 19MMHIS261e; approval date: 23 December 2019), and written informed consent was obtained from the parents or legal guardians of all participants.

Among preterm children, perinatal and neonatal clinical data were obtained from chart review. SGA was defined as a birth weight below the 10th percentile according to the Fenton growth chart [17]. Recorded neonatal morbidities included respiratory distress syndrome (RDS) requiring surfactant therapy, chronic lung disease (CLD), severe intraventricular hemorrhage (IVH), and retinopathy of prematurity (ROP).

### 2.2. Follow-Up at 5–6 Years of Age

#### 2.2.1. Anthropometric Measurements

Height and weight were measured using a digital scale, and age- and sex-adjusted z-scores were calculated according to Taiwanese Children Growth Charts [18]. Participants were then classified as underweight, normal weight, or overweight/obesity based on Taiwanese Children’s age- and sex-specific BMI criteria [18].

#### 2.2.2. Body Composition Measurements

Body composition was assessed using DXA (Horizon DXA System, APEX software version 13.6.0.5; Hologic Inc., Marlborough, MA, USA). All scans were performed by the same experienced pediatric densitometrist, who was blinded to the clinical status of the participants. Total lean mass and fat mass were normalized to height squared to derive lean mass index (LMI) and fat mass index (FMI). Trunk fat percentage and total body fat percentage were also measured.

#### 2.2.3. Biochemical Analysis

After an overnight fast of at least 8 h, blood samples were collected for the measurement of glucose, albumin, lipids, and insulin. Serum insulin was measured using an electrochemiluminescence immunoassay (ECLIA) on a Roche Cobas e801 analyzer (Roche Diagnostics, Mannheim, Germany). The intra-assay and inter-assay coefficients of variation were ≤2.1% and ≤3.2%, respectively. Children with acute illness or receiving medications known to affect glucose metabolism at the time of assessment were excluded. Insulin resistance was evaluated based on fasting insulin levels and the HOMA-IR, calculated as (fasting glucose [mg/dL] × insulin [µU/mL])/405.

### 2.3. Questionnaires

Parents completed self-administered questionnaires regarding their child’s current medication use and physical activity. Dietary intake was assessed using a 3-day dietary record covering two weekdays and one weekend day before the study visit. The records were reviewed by an experienced pediatric dietitian and analyzed using the Taiwan Food and Drug Administration Food Composition Database to estimate total energy and macronutrient intake, including protein, fat, saturated fat, fiber, and carbohydrates.

### 2.4. Growth Velocity

Anthropometric data, including weight and length/height, were obtained from medical records at birth, hospital discharge, and follow-up visits at corrected ages of 1 and 2 years and at 5–6 years. Interval-specific growth velocities for birth to discharge, discharge to 1 year, 1 to 2 years, and 2 to 5–6 years were calculated as the change in weight or length/height divided by the time elapsed between measurements.

### 2.5. Statistical Analysis

Continuous variables were compared using the independent *t* test or Mann–Whitney U test, as appropriate, and categorical variables were compared using the chi-square test or Fisher’s exact test. Among children born preterm, six prespecified clinically relevant outcomes, namely, trunk fat percentage, total fat percentage, LMI, FMI, insulin, and HOMA-IR, were compared across BMI categories (underweight, normal weight, and overweight/obesity) using general linear models adjusted for age and sex. Multivariable linear regression models were used to examine the associations between perinatal and clinical factors and body composition and insulin resistance outcomes, with covariates selected on the basis of clinical relevance. Spearman and partial correlation analyses were used to evaluate the associations between continuous perinatal or preschool variables and outcome measures. Partial correlation analyses were adjusted for age and sex. Statistical analyses were performed using IBM SPSS Statistics version 25.0, and a two-sided *p* value < 0.05 was considered statistically significant.

## 3. Results

### 3.1. Study Population

**Figure 1:**
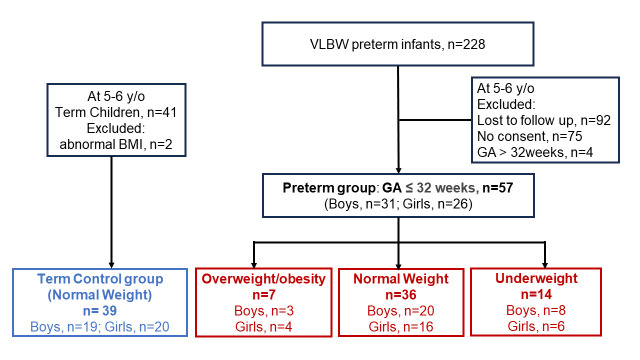
Flowchart illustrating the progression of study participants from birth to 5–6 years of age.

Figure 1 illustrates the flow of study participants from birth to 5–6 years of age. Among the 136 VLBW preterm children from our follow-up program, 61 (44.9%) consented to participate in both studies. After excluding four preterm children born after 32 weeks of gestation, 57 preterm children were included in the final analysis. The control group comprised 39 term-born children with BMI within the normal range. Sex distribution and the proportion of children born SGA were comparable between the preterm and term groups (Table 1). Within the preterm group, children were further classified as normal weight (*n* = 36), overweight/obesity (*n* = 7), and underweight (*n* = 14); five of the seven children in the overweight/obesity subgroup met the criteria for obesity.

### 3.2. Comparison Between Preterm and Term Children

At follow-up, preterm children were younger and had lower absolute height and weight than term-born controls. However, after standardization for age and sex, height and weight z-scores did not differ significantly between groups (Table 1). In DXA-based analyses, both the overall preterm cohort and the normal-weight preterm subgroup had lower total lean mass than term controls; however, LMI was similar after normalization to height. Other body composition measures, including FMI and fat distribution, were also comparable between groups after adjustment for age and sex (Table 1). In contrast, serum insulin levels and HOMA-IR were significantly higher in preterm children than in term controls (unadjusted *p* = 0.007 and *p* = 0.006, respectively; age- and sex-adjusted *p* = 0.003 and *p* = 0.004, respectively; Table 1). Similar findings were observed when the analysis was restricted to the normal-weight preterm subgroup, in which insulin and HOMA-IR remained higher than in term controls (adjusted *p* = 0.010 for both; Table 1).

Regarding dietary analysis at preschool age, fiber intake was the only variable that remained significantly lower in the preterm group than in the term group after adjustment for sex, age, and BMI (*p* < 0.001; Supplementary Table S1). No significant between-group differences were observed for total caloric intake, protein, fat, saturated fat, carbohydrate intake, or physical activity.

### 3.3. BMI Category and SGA Subgroup Analyses in Preterm Children

**Figure 2:**
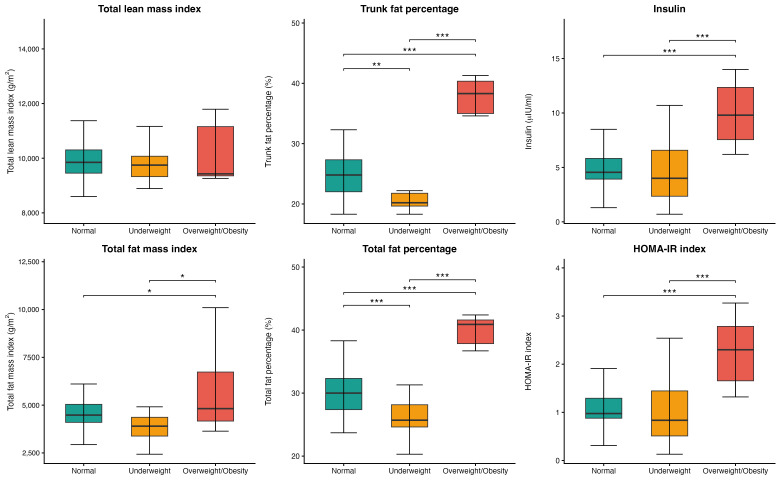
Comparison of body composition and insulin resistance indices across BMI categories in the preterm cohort at preschool age. Box plots show the distribution of trunk fat percentage, total body fat percentage, lean mass index (LMI), fat mass index (FMI), serum insulin, and homeostasis model assessment of insulin resistance (HOMA-IR) among preterm children classified as underweight, normal weight, and overweight/obesity. All comparisons were performed using general linear models adjusted for age and sex. The overweight/obesity group had significantly higher values than both the normal weight and underweight groups across most adiposity and insulin resistance measures, including trunk fat percentage, total body fat percentage, FMI, insulin, and HOMA-IR, but not LMI. The underweight group had significantly lower trunk and total body fat percentages than the normal weight group, whereas FMI, insulin, and HOMA-IR did not differ significantly between these two groups. Asterisks above the brackets indicate the significance of pairwise between-group differences: * adjusted *p* < 0.05; ** adjusted *p* < 0.01; *** adjusted *p* < 0.001.

Within the preterm cohort, age- and sex-adjusted analyses showed significant differences across BMI categories in trunk fat percentage, total fat percentage, FMI, insulin, and HOMA-IR (overall *p* < 0.001, *p* < 0.001, *p* = 0.022, *p* < 0.001, and *p* < 0.001, respectively), whereas LMI did not differ significantly (*p* = 0.194) (Figure 2). Compared with the normal-weight group, the overweight/obesity group had higher trunk fat percentage, total fat percentage, FMI, insulin, and HOMA-IR (adjusted *p* < 0.001, *p* < 0.001, *p* = 0.046, *p* < 0.001, and *p* < 0.001, respectively). A similar pattern was observed when the overweight/obesity group was compared with the underweight group (adjusted *p* < 0.001, *p* < 0.001, *p* = 0.020, *p* < 0.001, and *p* < 0.001, respectively). By contrast, the underweight group had lower trunk fat percentage (adjusted *p* = 0.003) and total fat percentage (adjusted *p* < 0.001) than the normal-weight group, whereas FMI, insulin, and HOMA-IR did not differ significantly between these two groups (adjusted *p* = 0.253, *p* = 0.637, and *p* = 0.605, respectively).

When preterm children were stratified by SGA status, those born SGA had lower weight, height, and BMI z-scores than those born non-SGA (*p* = 0.019, *p* = 0.006, and *p* = 0.012, respectively). They also had lower trunk fat percentage and total body fat percentage than their non-SGA counterparts (adjusted *p* < 0.001 and *p* = 0.005, respectively). No other significant differences in body composition measures were observed according to SGA status.

### 3.4. Perinatal and Clinical Factors Associated with Outcomes in Preterm Children

Male sex was significantly associated with a lower total fat percentage (β = −3.17, 95% CI −5.86 to −0.47, *p* = 0.021), whereas no other perinatal or clinical factors were significantly associated with body composition parameters or insulin resistance. This finding remained unchanged when CLD was replaced with other neonatal morbidities, including RDS, IVH, and ROP, in separate models (Supplementary Table S2).

### 3.5. Sex-Specific Analyses

When the comparison between term controls and the normal-weight preterm subgroup was stratified by sex, sex-specific differences in body composition were observed (Table 2). In the term group, boys had a higher LMI than girls (unadjusted *p* < 0.001; age-adjusted *p* = 0.003), whereas this difference was not observed in the normal-weight preterm subgroup. In both the term and preterm groups, girls had higher trunk and total body fat percentages than boys (term: adjusted *p* < 0.001 for both; preterm: adjusted *p* = 0.034 and *p* = 0.030, respectively). Serum biochemical markers, including HOMA-IR, did not differ significantly by sex.

### 3.6. Correlation Analyses and Growth Trajectories

Across the entire cohort, gestational age and birth weight showed weak but significant inverse correlations with serum insulin and HOMA-IR after adjustment for age and sex, whereas no corresponding associations were observed with body composition measures (Table 3). In contrast, weight at 5–6 years of age was significantly associated with LMI, FMI, trunk and total body fat percentages, and insulin resistance in the overall cohort (Table 3). Dietary intake was not associated with body composition measures or insulin resistance (Supplementary Table S3).

With respect to postnatal growth (Table 3), weight gain velocity between 2 and 5–6 years of age remained significantly associated with trunk and total body fat percentages, as well as serum insulin levels and HOMA-IR, after adjustment for age and sex. By contrast, weight gain during earlier intervals and all height growth velocities were not significantly associated with these outcomes.

## 4. Discussion

In this study, children born before 33 weeks of gestation with VLBW had higher insulin levels and HOMA-IR at 5–6 years of age than term-born controls, despite comparable body composition after adjustment for age and sex. Within the preterm cohort, overweight/obesity was associated with greater adiposity and higher insulin resistance. In addition, weight gain during later childhood, rather than earlier growth, was associated with adiposity and metabolic markers. Together, these findings suggest that metabolic alterations may already be detectable in preschool-aged children born very preterm with VLBW, even in the absence of marked differences in overall body composition compared with that seen in term-born peers.

Early postnatal fat accretion is a physiological adaptation to extrauterine life, whereas excess adiposity, particularly trunk fat, has been associated with increased susceptibility to cardiovascular diseases and insulin resistance in preterm infants and adolescents [19]. Lean mass is also essential for organ development and metabolic function [20,21], as skeletal muscle is a major site of glucose uptake, and reduced muscle mass has been associated with insulin resistance [22]. Body composition assessment provides important information beyond conventional anthropometric measures and may help characterize the quality of growth after preterm birth. Previous studies comparing body composition between preschool-aged preterm and term-born children have reported inconsistent findings. Consistent with our results, Darendeliler et al. reported no significant difference in FMI between preterm and term-born children at 4–5 years of age [23]. Similarly, Huke et al. found no evidence of increased fat mass or intra-abdominal adiposity in preterm children aged 5–7 years [10]. In contrast, several studies have reported significantly lower lean mass or fat free mass in preterm children compared with that seen in term counterparts at school age [8,12]. In our cohort, however, adjusted measures of fat and lean mass were largely comparable between groups, suggesting that differences in body composition are not yet detectable at this age. These discrepancies may reflect differences in study populations, ages at assessment, and adjustment for body size. Although DXA provides more detailed information on body composition than conventional anthropometric measurements, its routine use in clinical follow-up may be limited by cost and availability. Therefore, standard anthropometric monitoring remains the cornerstone of follow-up in most settings, while DXA may be useful in selected children when more detailed body composition assessment is clinically indicated.

Both insulin levels and HOMA-IR values have been validated as reliable indicators of insulin resistance in children [24]. Over time, insulin resistance may contribute to metabolic syndrome. By demonstrating higher fasting insulin and HOMA-IR in children born very preterm with VLBW despite generally similar DXA-derived body composition, this study contributes to the limited preschool-age literature on metabolic health in preterm populations. This pattern suggests that altered metabolic status may be present even in the absence of clear differences in overall body composition at this age, and may indicate that metabolic alterations precede detectable changes in adiposity. However, although fasting insulin and HOMA-IR were significantly higher in the preterm group, the absolute values remained within the accepted normal range for preschool-aged children. Therefore, these findings should not be interpreted as evidence of overt clinical insulin resistance. Rather, they may represent early metabolic alterations, the long-term clinical significance of which remains uncertain. Whether these differences translate into increased cardiometabolic risk later in life requires further longitudinal follow-up. Although the underlying causes remain unclear, several mechanisms have been proposed to contribute to insulin resistance in individuals born preterm, including accelerated postnatal growth, altered body composition, chronic inflammation, and early nutritional exposures. However, these pathways were not directly assessed in the present study. However, in contrast to our findings, some studies have reported no significant differences in insulin levels or HOMA-IR indices among preterm children [25,26]. Therefore, further large-scale longitudinal studies are warranted to validate our results.

Overweight or obesity are associated with higher adipokine levels, increased fat mass, and abnormal fat distribution, all of which are linked to insulin resistance. Consistent with previous studies, we found that BMI category was closely associated with adiposity and insulin resistance within the preterm cohort [19]. Children with overweight or obesity had higher trunk fat percentage, total fat percentage, FMI, insulin levels, and HOMA-IR than both the normal-weight and underweight groups, whereas LMI did not differ significantly across BMI categories. These findings suggest that variation in BMI status at preschool age in children born preterm is more strongly related to fat accretion than to lean tissue. They also underscore the importance of early weight management in preschool-aged children and highlight the need for close monitoring of insulin resistance from an early stage in children with overweight or obesity.

Our findings suggest that the long-term metabolic implications of postnatal growth in children born preterm may depend not only on the occurrence of catch-up growth but also on its timing. Previous studies have shown that weight gain after hospital discharge or during early childhood may be associated with greater fat accumulation, and less favorable metabolic profiles later in life [15,16]. In line with these observations, weight gain velocity during hospitalization was not associated with preschool body composition in our cohort, whereas weight gain velocity between 2 and 5–6 years was positively associated with trunk fat percentage, total fat percentage, insulin levels, and HOMA-IR. By contrast, linear growth was not associated with these outcomes. Although some studies have reported no association between growth trajectories and later body composition [9,27], our results suggest that later childhood may represent a particularly important period during which accelerated weight gain is related to subsequent adiposity and metabolic vulnerability in children born preterm.

Sex also plays a significant role in body composition. Girls had higher trunk and total body fat percentages than boys in both the term and preterm groups, suggesting that sexual dimorphism in fat distribution may already be apparent by 5–6 years of age. In addition, male sex was independently associated with a lower total fat percentage in multivariable models. These observations are consistent with known sex-related differences in body composition and suggest that sex should be considered when interpreting metabolic and body composition outcomes in this population.

Early life nutrition plays a crucial role in shaping body composition in preterm infants, acting as a key factor in metabolic programming [28]. However, some studies have found no significant impact of macronutrient intake on body composition [29]. Furthermore, there is conflicting evidence regarding the protective role of breastfeeding in body composition [30,31,32]. In our study, we did not observe any significant association between breast milk feeding during hospitalization or nutritional intake at 5–6 years of age and body composition. However, interpretation of these findings is limited because detailed nutritional exposure from birth through infancy was not available, and most preterm infants in our cohort received human milk during hospitalization, reducing variability in early feeding exposure.

Early identification of risk factors is crucial to reducing the risk of chronic diseases in this vulnerable population. Among these factors, nutritional support and metabolic management during NICU hospitalization may play important roles in long-term metabolic programming. Adequate provision of amino acids and protein during early life, together with careful monitoring of glucose homeostasis and avoidance of excessive hyperglycemia, are considered important components of optimal nutritional care for preterm infants. Previous studies have indicated a relationship between necrotizing enterocolitis and impaired bone health [33]. However, no neonatal morbidity was significantly associated with body composition or insulin resistance in our models. A recent meta-analysis reported that individuals born SGA had lower fat mass and lean mass than their non-SGA counterparts [13]. In our study, preterm children born SGA had lower fat distribution measures but did not differ in insulin resistance, consistent with prior studies [34]. The small number of SGA children may have limited our ability to detect subtle associations. In addition, although some evidence indicates that physical activity promotes lean mass gain in preterm infants [35], this association was not observed in our study.

This study has several strengths, including prospective follow-up of preterm participants. We employed DXA, the most reliable and accurate noninvasive technique for assessing body composition in children [7,36]. Nevertheless, several limitations should be considered. First, this study has a relatively small sample size, particularly in subgroup analyses, owing to its single-center design. Power analysis yielded statistical power estimates of 0.306 for trunk fat percentage and 0.651 for HOMA-IR, indicating limited to moderate power to detect differences. In particular, the overweight/obesity subgroup comprised only seven children; therefore, findings from subgroup analyses should be considered exploratory and interpreted with caution. Some non-significant associations may reflect limited statistical power rather than the absence of a true association. Second, although only a subset of eligible VLBW children participated in follow-up, participants and non-participants did not differ significantly in available baseline perinatal characteristics (Supplementary Table S4). These findings suggest that the enrolled cohort was broadly representative of the eligible VLBW population with respect to the available baseline clinical characteristics. Nevertheless, the potential influence of unmeasured factors related to follow-up participation cannot be excluded. Third, as the study population consisted of preterm children born before 33 weeks of gestation in Taiwan, the findings may not be generalizable to other populations. In addition, restricting the control group to term-born children with normal BMI may introduce selection bias and limit the representativeness of the study. The term controls likely represent a metabolically healthier reference group than the general pediatric population. Therefore, comparisons involving adiposity and insulin resistance should be interpreted with caution, as the observed differences may not fully reflect those seen in a population-representative control cohort. Future studies should include control groups reflecting the broader BMI distribution of healthy term-born children. Furthermore, detailed longitudinal nutritional data from infancy and early childhood were unavailable. Therefore, the potential contribution of early nutritional programming to later body composition and metabolic outcomes could not be fully evaluated. Finally, the relatively short observation period, with evaluations limited to 5–6 years, may not have detected changes in body composition and insulin resistance that could have emerged or resolved later. Therefore, large-scale longitudinal follow-up studies are required to confirm our findings.

## 5. Conclusions

In conclusion, preschool-aged children born before 33 weeks of gestation with VLBW exhibited higher fasting insulin levels and HOMA-IR at preschool age, despite generally comparable DXA-derived body composition relative to term-born controls. Within the preterm cohort, overweight/obesity and greater weight gain from 2 to 5–6 years of age were associated with increased adiposity and higher insulin resistance markers. Clinically, our results support long-term follow-up strategies that extend beyond routine growth assessment to include monitoring of metabolic health, while nutritional management in the NICU and early childhood should balance adequate growth promotion with avoidance of excessive weight gain. From a public health perspective, structured long-term surveillance and early preventive interventions for children born very preterm, particularly those with overweight or obesity, may help identify those at increased metabolic risk. Larger longitudinal studies are required to determine whether these early metabolic alterations translate into adverse cardiometabolic outcomes later in life.

## Figures and Tables

**Table 1:** Comparison of anthropometry, body composition, and serum biochemistry data between preterm and term groups and within the preterm normal-weight subgroup.

	Term Group (*n* = 39)	Preterm Group (*n* = 57)	*p* Value	Preterm with Normal Weight Subgroup (*n* = 36)	*p* Value
GA (weeks)	39 (38–39)	28 (26–30)	**<0.001**	28 (26–29)	**<0.001**
BW (gm)	2980 (2781–3354)	1078 (795–1255)	**<0.001**	1014 (807–1258)	**<0.001**
Boys (%)	49	54	0.585	56	0.554
SGA (%)	15	14	0.854	6	0.265
**At 5–6 years old**					
Anthropometry					
Age (years)	5.7 (5.5–6.3)	5.4 (5.2–5.5)	**<0.001**	5.4 (5.2–5.4)	**<0.001**
Height (cm)	114.1 (111.2–118.2)	111.0 (108.0–114.0)	**0.001**	111.2 (108.0–113.4)	**0.001**
Height z-score	0.09 (−0.23–0.49)	−0.29 (−0.72–0.59)	0.171	−0.06 (−0.62–0.61)	0.351
Weight (kg)	19.4 (18.1–20.9)	18.0 (16.5–20.2)	**0.007**	18.2 (17.4–19.3)	**0.015**
Weight z-score	−0.12 (−0.66–0.22)	−0.43 (−1.26–0.39)	0.213	−0.29 (−0.66–0.30)	0.645
BMI	14.8 (14.1–15.7)	14.8 (13.6–15.7)	0.476	15.1 (14.3–15.7)	0.671
BMI z-score	−0.44 (−1.07–0.27)	−0.47 (−1.65–0.32)	0.403	−0.17 (−0.90–0.27)	0.795
Body Composition *					
Total lean mass (g)	13,296 (11,989–14,633)	12,311 (11,409–13,377)	**0.005**	12,603 (11,669–13,306)	0.087
Total lean mass index (g/m^2^)	10,141 (9569–10,602)	9843 (9427–10,352)	0.235	9849 (9453–10,304)	0.092
Total fat mass (g)	5624 (5338–6500)	5369 (4509–6314)	0.809	5613 (5060–6215)	0.786
Total fat mass index (g/m^2^)	4668 (4030–5045)	4461 (3828–4980)	0.876	4480 (4100–5042)	0.314
Trunk fat (%)	23.2 (21.2–26.1)	23.7 (20.6–27.7)	0.179	24.8 (22.0–27.3)	0.337
Total body fat (%)	29.3 (26.8–32.1)	29.4 (26.2–32.4)	0.409	30.0 (27.4–32.3)	0.538
Biochemistry *					
Glucose (mg/dL)	89 (83–91)	89 (85–92)	0.219	90 (85–92)	0.178
Albumin (g/dL)	4.7 (4.6–5.0)	4.8 (4.7–4.9)	0.103	4.8 (4.7–5.0)	0.262
Cholesterol (mg/dL)	171 (158–192)	171 (164–245)	0.940	172 (161–238)	0.643
Triglyceride (mg/dL)	40 (35–53)	43 (34–53)	0.935	43 (35–50)	0.849
LDL (mg/dL)	98 (84–110)	105 (84–112)	0.488	106 (88–113)	0.578
HDL (mg/dL)	63 (54–69)	60 (51–68)	0.303	58 (50–66)	0.174
Insulin (μU/mL)	3.8 (2.3–5.0)	5.0 (3.4–6.9)	**0.003**	4.6 (3.9–5.8)	**0.010**
HOMA-IR index	0.85 (0.50–1.10)	1.07 (0.69–1.52)	**0.004**	0.98 (0.88–1.29)	**0.010**

GA, gestational age; BW, birth weight; SGA, small for gestational age; LDL, Low-Density Lipoprotein; HDL, High-Density Lipoprotein; HOMA-IR, Homeostasis Model Assessment of Insulin Resistance. Preterm all group and Preterm normal BMI group: statistical results were compared with the term group. * *p* values were derived from multivariable models adjusted for age and sex.

**Table 2:** Sex differences in anthropometry, main body composition, and insulin resistance in term group and preterm normal-weight subgroup.

	Term Group			Preterm with Normal Weight Subgroup		
	Boys (*n* = 19)	Girls (*n* = 20)	*p* Value	Boys (*n* = 20)	Girls (*n* = 16)	*p* Value
GA (weeks)	39 (38–40)	39 (38–39)	0.835	28 (27–29)	27 (25–29)	0.789
BW (gm)	2900 (2766–3312)	2990 (2896–3375)	0.396	1167 (966–1271)	873 (733–1110)	0.062
SGA (%)	26	5	0.258	5	6	0.962
At 5–6 years old						
Anthropometry						
Age (years)	5.7 (5.4–6.3)	5.7 (5.5–6.2)	0.945	5.4 (5.2–5.4)	5.4 (5.3–5.4)	0.741
Height z-score	−0.11 (−0.23–0.38)	0.15 (−0.15–0.85)	0.365	−0.08 (−0.68–0.74)	−0.06 (−0.48–0.32)	0.789
Weight z-score	−0.10 (−0.74–0.46)	−0.28 (−0.63–0.06)	0.550	−0.35 (−0.61–0.11)	−0.26 (−0.96–0.30)	0.863
BMI z-score	0.05 (−0.95–0.83)	−0.55 (−1.05–−0.06)	0.214	−0.23 (−1.09–0.27)	−0.05 (−0.71–0.28)	0.648
Body Composition *						
Lean mass index (g/m^2^)	10,510 (10,225–11,395)	9778 (9394–10,113)	0.003	9893 (9647–10,304)	9808 (9401–10,069)	0.387
Fat mass index (g/m^2^)	4587 (3891–5080)	4726 (4283–5039)	0.830	4505 (4177–5061)	4480 (3828–4991)	0.972
Trunk fat (%)	21.2 (20.0–23.3)	24.7 (22.7–27.8)	<0.001	23.1 (20.6–26.7)	25.9 (23.4–27.9)	0.034
Total body fat (%)	27.3 (26.1–28.3)	31.1 (29.0–32.9)	<0.001	28.9 (26.1–31.6)	31.9 (29.4–33.3)	0.030
Insulin Resistance *						
Insulin (μU/mL)	3.2 (2.1–5.2)	4.1 (2.7–4.5)	0.608	4.3 (3.4–5.0)	5.4 (4.0–6.5)	0.190
HOMA-IR index	0.69 (0.46–1.17)	0.87 (0.55–1.02)	0.467	0.95 (0.79–1.08)	1.17 (0.89–1.42)	0.231

GA, gestational age; BW, birth weight; SGA, small for gestational age; HOMA-IR, Homeostasis Model Assessment of Insulin Resistance. * *p* values were derived from multivariable models adjusted for age.

**Table 3:** Association between BMI, growth velocities, key body composition, and insulin resistance in preschool-aged children.

	Lean Mass Index (g/m^2^)	Fat Mass Index (g/m^2^)	Trunk Fat (%)	Total Body Fat (%)	Insulin (μU/mL)	HOMA-IR Index
**All participants (*n* = 96)**						
GA (weeks)	r = 0.161, *p* = 0.122	r = 0.035, *p* = 0.738	r = −0.088, *p* = 0.402	r = −0.074, *p* = 0.480	**r = −0.266 (−0.45 to −0.07),** ***p* = 0.010**	**r = −0.264 (−0.44 to −0.07),** ***p* = 0.010**
BW (gm)	r = 0.036, *p* = 0.729	r = 0.134, *p* = 0.199	r = 0.031, *p* = 0.765	r = 0.052, *p* = 0.619	**r = −0.285 (−0.46 to −0.09),** ***p* = 0.005**	**r = −0.292 (−0.47 to −0.10),** ***p* = 0.004**
Age (year)	r = 0.100, *p* = 0.333	r = −0.029, *p* = 0.779	r = −0.129, *p* = 0.209	r = −0.160, *p* = 0.119	r = −0.066, *p* = 0.523	r = −0.059, *p* = 0.570
Height (cm)	r = 0.102, *p* = 0.327	r = 0.125, *p* = 0.231	r = 0.099, *p* = 0.344	r = 0.164, *p* = 0.114	r = −0.040, *p* = 0.700	r = −0.021, *p* = 0.842
Weight (kg)	**r = 0.222 (0.02 to 0.41),** ***p* = 0.032**	**r = 0.282 (0.09 to 0.46),** ***p* = 0.006**	**r = 0.363 (0.17 to 0.53),** ***p* < 0.001**	**r = 0.436 (0.26 to 0.58),** ***p* < 0.001**	**r = 0.218 (0.02 to 0.41),** ***p* = 0.035**	**r = 0.228 (0.03 to 0.42),** ***p* = 0.027**
**Growth Velocities in Preterm groups (*n* = 57)**						
Birth–discharge weight growth (g/d)	r = −0.122, *p* = 0.381	r = 0.147, *p* = 0.287	r = 0.063, *p* = 0.649	r = 0.036, *p* = 0.793	r = −0.271, *p* = 0.160	r = −0.255, *p* = 0.063
Discharge–1y weight growth (kg/month)	r = 0.137, *p* = 0.317	r = 0.273, *p* = 0.160	r = 0.410, *p* = 0.052	r = 0.352, *p* = 0.067	r = 0.008, *p* = 0.953	r = 0.010, *p* = 0.940
1–2 y weight growth (kg/month)	r = −0.009, *p* = 0.950	r = −0.061, *p* = 0.657	r = −0.005, *p* = 0.973	r = 0.023, *p* = 0.867	r = −0.030, *p* = 0.827	r = −0.022, *p* = 0.876
2y–5–6y weight growth (kg/year)	r = 0.182, *p* = 0.183	**r = 0.266 (0.00 to 0.50),** ***p* = 0.060**	**r = 0.541 (0.32 to 0.71),** ***p* < 0.001**	**r = 0.556 (0.34 to 0.72),** ***p* < 0.001**	**r = 0.469 (0.23 to 0.65)** **,** ***p* = 0.004**	**r = 0.472 (0.24 to 0.66),** ***p* = 0.004**

Values are shown as r and *p*. Except for the Age row, all correlations are partial Spearman correlations adjusted for age and sex. Significant correlation coefficients are accompanied by 95% confidence intervals. GA, gestational age; BW, birth weight.

## Data Availability

The data presented in this study are available from the corresponding author upon reasonable request. The data are not publicly available due to privacy and ethical restrictions involving pediatric participants.

## Supplementary Materials

The following supporting information can be downloaded at: https://www.mdpi.com/article/10.3390/diagnostics16131991/s1, Table S1: Comparison of Dietary Intake and Physical Activity Between the Term and Preterm Groups; Table S2: Multivariable Models for Body Composition and Insulin Resistance Outcomes in the Preterm Group; Table S3: Associations of Dietary Intake with Body Composition and Insulin Resistance in Preschool-Aged Children; Table S4: Comparison of baseline perinatal characteristics between enrolled and non-enrolled eligible VLBW preterm infants.

## Abbreviations

The following abbreviations are used in this manuscript:

BMI	Body mass index
BW	Birth weight
CLD	Chronic lung disease
DXA	Dual-energy X-ray absorptiometry
FMI	Fat mass index
GA	Gestational age
HDL	High-density lipoprotein
HOMA-IR	Homeostasis Model Assessment of Insulin Resistance
IVH	Intraventricular hemorrhage
LDL	Low-density lipoprotein
LMI	Lean mass index
RDS	Respiratory distress syndrome
ROP	Retinopathy of prematurity
SGA	Small for gestational age
VLBW	Very low birth weight
