# Supplementary material for: DXA-Derived Body Composition and Insulin Resistance at Preschool Age in Very-Low-Birth-Weight Preterm Infants: A Prospective Cohort Study
Source: Diagnostics (Basel). 2026 Jun 26;16(13):1991. doi: 10.3390/diagnostics16131991 (PMC13360083; doi:10.3390/diagnostics16131991)
Supplement: Supplementary file 1 [file diagnostics-16-01991-s001.zip › diagnostics-4306884-supplementary.pdf]

Supplementary Table S1. Comparison of Dietary Intake and Physical Activity Between the Term and Preterm Groups

Variable	Term group	Preterm group	Adjusted β	95% CI	p value
**Calories (kcal)**	1432.37 ± 256.57	1330.92 ± 258.14	-81.74	-197.18 to 33.71	0.163
**Protein (g)**	49.04 ± 11.37	46.90 ± 10.12	-0.68	-5.24 to 3.88	0.767
**Fat (g)**	56.40 ± 13.01	51.71 ± 13.39	-3.17	-8.91 to 2.57	0.275
**Carbohydrate (g)**	183.09 ± 37.25	169.78 ± 38.01	-13.62	-30.76 to 3.51	0.118
**Fiber (g)**	8.38 ± 2.88	6.49 ± 2.21	-2.28	-3.38 to -1.17	**<0.001**
**Physical activity (hours/week)**	3.99 ± 1.92	3.76 ± 1.98	0.18	-0.64 to 1.00	0.663

Note. Data are presented as mean ± SD. Adjusted β represents the difference between the preterm and term groups after adjustment for age, sex, and BMI.

Supplementary Table S2. Multivariable Models for Body Composition and Insulin Resistance Outcomes in the Preterm Group

Predictor	Total lean index (g/m^2)	Total fat index (g/m^2)	Trunk fat (%)	Total fat (%)	Insulin	HOMA-IR index
**Age at 5 years (per year)**	β = 15.03<br>95% CI, -596.92 to 626.97<br>p = 0.962	β = 621.13<br>95% CI, -879.55 to 2121.81<br>p = 0.417	β = -1.32<br>95% CI, -4.86 to 2.22<br>p = 0.464	β = -1.30<br>95% CI, -4.02 to 1.43<br>p = 0.351	β = 0.09<br>95% CI, -2.45 to 2.63<br>p = 0.945	β = 0.06<br>95% CI, -0.55 to 0.67<br>p = 0.844
**Male sex**	β = 385.23<br>95% CI, -146.45 to 916.90<br>p = 0.156	β = 230.92<br>95% CI, -569.09 to 1030.93<br>p = 0.572	β = -3.09<br>95% CI, -6.24 to 0.07<br>p = 0.055	β = -3.17<br>95% CI, -5.86 to -0.47<br>**p = 0.021**	β = -0.15<br>95% CI, -2.07 to 1.78<br>p = 0.882	β = -0.02<br>95% CI, -0.46 to 0.42<br>p = 0.939
**Maternal diabetes**	β = -75.03<br>95% CI, -778.39 to 628.33<br>p = 0.834	β = -336.00<br>95% CI, -1371.28 to 699.27<br>p = 0.525	β = -1.96<br>95% CI, -6.29 to 2.38<br>p = 0.377	β = -1.70<br>95% CI, -5.79 to 2.38<br>p = 0.414	β = -1.83<br>95% CI, -3.83 to 0.17<br>p = 0.072	β = -0.42<br>95% CI, -0.87 to 0.04<br>p = 0.072
**Human milk**	β = -459.88<br>95% CI, -970.12 to 50.37<br>p = 0.077	β = 232.52<br>95% CI, -578.76 to 1043.81<br>p = 0.574	β = 0.44<br>95% CI, -2.74 to 3.62<br>p = 0.784	β = 0.97<br>95% CI, -1.98 to 3.91<br>p = 0.521	β = -0.98<br>95% CI, -2.73 to 0.76<br>p = 0.269	β = -0.22<br>95% CI, -0.62 to 0.18<br>p = 0.286

**CLD**	β = -117.13<br>95% CI, -655.87 to 421.62<br>p = 0.670	β = 48.73<br>95% CI, -723.90 to 821.37<br>p = 0.902	β = -2.12<br>95% CI, -5.31 to 1.07<br>p = 0.192	β = -1.69<br>95% CI, -4.56 to 1.17<br>p = 0.247	β = -0.73<br>95% CI, -2.62 to 1.16<br>p = 0.450	β = -0.17<br>95% CI, -0.60 to 0.26<br>p = 0.438

Note. Values are regression coefficients (β), 95% confidence intervals (CIs), and p values from separate multivariable linear regression models. All models were fitted in the preterm group only. CLD, chronic lung disease.

Supplementary Table S3. Associations of Dietary Intake with Body Composition and Insulin Resistance in Preschool-Aged Children

	Total lean index	Total fat index	Trunk fat %	Total fat %	Insulin	HOMA-IR index
**Calories (kcal)**	r = -0.124<br>p = 0.250	r = -0.156<br>p = 0.148	r = -0.062<br>p = 0.565	r = -0.053<br>p = 0.622	r = -0.096<br>p = 0.372	r = -0.091<br>p = 0.397
**Protein (g)**	r = -0.035<br>p = 0.748	r = -0.145<br>p = 0.179	r = 0.064<br>p = 0.552	r = 0.057<br>p = 0.601	r = 0.012<br>p = 0.912	r = 0.014<br>p = 0.893
**Fat (g)**	r = -0.181<br>p = 0.092	r = -0.120<br>p = 0.267	r = -0.018<br>p = 0.868	r = -0.005<br>p = 0.965	r = -0.033<br>p = 0.757	r = -0.033<br>p = 0.759
**Carbohydrate (g)**	r = -0.077<br>p = 0.475	r = -0.146<br>p = 0.175	r = -0.144<br>p = 0.180	r = -0.128<br>p = 0.236	r = -0.178<br>p = 0.096	r = -0.171<br>p = 0.112
**Fiber (g)**	r = -0.000<br>p = 0.997	r = -0.012<br>p = 0.914	r = -0.111<br>p = 0.304	r = -0.125<br>p = 0.248	r = -0.032<br>p = 0.765	r = -0.050<br>p = 0.647

Note. Values are partial correlation coefficients (r) with corresponding p values, adjusted for age and sex. HOMA-IR, homeostasis model assessment of insulin resistance.

Supplementary Table S4. Comparison of baseline perinatal characteristics between enrolled and non-enrolled eligible VLBW preterm infants

	Enrolled group N=57	Not enrolled Group N=79	p-value
Antenatal steroids (%)	90	87	0.554
Preeclampsia (%)	28	27	0.832
PROM >18hours (%)	37	39	0.814
Cesarean section (%)	68	77	0.177
Singleton birth (%)	81	79	0.680
GA, week (mean± SD)	28.4 ± 3.0	28.6 ± 3.1	0.712
BW, g (mean± SD)	1065 ± 272	1055 ± 278	0.694
Small for gestational age (%)	31	32	0.534
Male gender (%)	58	46	0.167
Apgar score at 1 min (mean± SD)	6.5 ± 1.9	6.4 ± 2.0	0.525
Apgar score at 5 min (mean± SD)	8.2 ± 1.3	8.1 ± 1.7	0.250
RDS need surfactant (%)	21	32	0.156
BPD (%)	48	46	0.920
Sepsis (%)	16	14	0.843
PDA need treatment (%)	28	34	0.222
ROP ≥ stage 3 (%)	14	15	0.640
Severe IVH (%)	7	3	0.296

Abbreviations: BW, birth weight; BPD, bronchopulmonary dysplasia; GA, gestational age; IVH, intraventricular hemorrhage; PDA, patent ductus arteriosus; PROM, prolonged rupture of membranes; RDS, respiratory distress syndrome; ROP, retinopathy of prematurity.
